# Increased expression of connexin 43 in a mouse model of spinal motoneuronal loss

**DOI:** 10.18632/aging.103561

**Published:** 2020-06-24

**Authors:** Federica Maria Spitale, Nunzio Vicario, Michelino Di Rosa, Daniele Tibullo, Michele Vecchio, Rosario Gulino, Rosalba Parenti

**Affiliations:** 1 Department of Biomedical and Biotechnological Sciences, Section of Physiology, University of Catania, Catania 95123, Italy; 2 Department of Biomedical and Biotechnological Sciences, Section of Anatomy, Histology and Movement Sciences, University of Catania, Catania 95123, Italy; 3 Department of Biomedical and Biotechnological Sciences, Section of Biochemistry, University of Catania, Catania 95123, Italy; 4 Department of Biomedical and Biotechnological Sciences, Section of Pharmacology, University of Catania, Catania 95123, Italy; 5 Rehabilitation Unit, “AOU Policlinico Vittorio Emanuele”, University of Catania, Catania 95123, Italy

**Keywords:** ALS, neurodegeneration, neuronal loss, astrocyte, gap junction

## Abstract

Amyotrophic lateral sclerosis (ALS) is one of the most common motoneuronal disease, characterized by motoneuronal loss and progressive paralysis. Despite research efforts, ALS remains a fatal disease, with a survival of 2-5 years after disease onset. Numerous gene mutations have been correlated with both sporadic (sALS) and familiar forms of the disease, but the pathophysiological mechanisms of ALS onset and progression are still largely uncertain. However, a common profile is emerging in ALS pathological features, including misfolded protein accumulation and a cross-talk between neuroinflammatory and degenerative processes. In particular, astrocytes and microglial cells have been proposed as detrimental influencers of perineuronal microenvironment, and this role may be exerted via gap junctions (GJs)- and hemichannels (HCs)-mediated communications. Herein we investigated the role of the main astroglial GJs-forming connexin, Cx43, in human ALS and the effects of focal spinal cord motoneuronal depletion onto the resident glial cells and Cx43 levels. Our data support the hypothesis that motoneuronal depletion may affect glial activity, which in turn results in reactive Cx43 expression, further promoting neuronal suffering and degeneration.

## INTRODUCTION

Amyotrophic lateral sclerosis (ALS) is a progressive neurodegenerative disease that affects upper and lower motoneurons [1, 2]. Although the main ALS hallmark is motoneuronal loss due to motoneuron vulnerability, resident glial cells play a crucial role in ALS pathogenesis. In particular, during the disease progression, a robust neuroinflammation, glial activation and misfolded protein accumulation can be observed, together driving progressive neuronal loss and persistent disabilities [3, 4]. Recent evidence on neurodegenerative/inflammatory disorders have highlighted a key role of neuroglial cross-talk, which substantially contributes to neuronal suffering and degeneration [3, 4].

Gap junctions (GJs) are characterized by the juxtaposition of two hemichannels (HCs) of adjacent cells, and allow the exchange of ions, metabolites, and other mediators < 1 kDa between intracellular fluids (i.e. GJs-mediated intercellular communication) or between intracellular and extracellular compartment (i.e. HCs-mediated communication) [5, 6]. GJs are aggregates in defined plasma membrane regions of adjacent cells forming the so-called GJs plaques, in which GJs are rapidly assembled, disassembled or remodelled [6]. Previous evidence demonstrated that connexins (Cxs), the core GJs- and HCs-forming proteins, exert a prominent role in maintaining physiological functions and promoting reactive activation of glial cells [7]. Indeed, previous reports on transgenic mouse models of ALS, showed an early Cx43-reactive expression on spinal cord microenvironment. This evidence was also observed in aging and in major neurodegenerative disorders, including spinal cord injury and Alzheimer’s disease [8–10]. It seems likely that ALS has a focal onset in the central nervous system, where microenvironmental conditions are particularly hostile and mediate neurodegeneration spread and progression [2, 11, 12]. Thus, we developed a mouse model of focal removal of lumbar spinal cord motoneurons using retrograde suicide transport of saporin, conjugated to cholera toxin-B subunit (CTB-Sap) [13, 14].

Herein we investigated Cx43, the most abundant GJs- and HCs-forming protein of the central nervous system, and its possible role in human ALS, as well as in the CTB-Sap model [13, 14]. We have shown that Cx43-reactive expression may represent the biological substrate underlying reactive glial activation and neuronal suffering in neurodegenerative diseases.

## RESULTS

### Correlation between GJA1 and GFAP in human ALS

**Figure 1:**
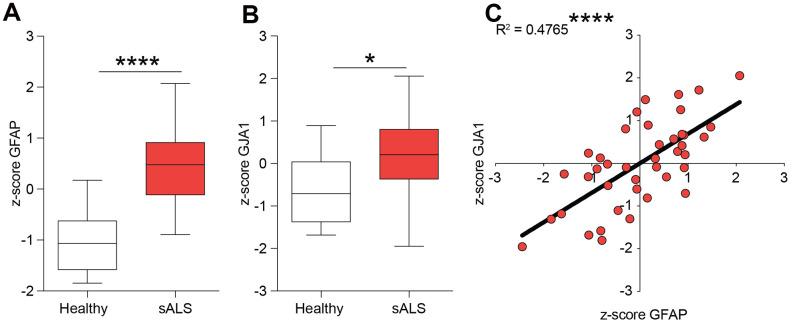
**Expression levels of GFAP and GJA1 encoding for Cx43 in human sALS biopsies.** (**A**, **B**) mRNA expression levels of GFAP (**A**) and GJA1 (**B**) in the central nervous system of sALS patients versus healthy control levels. Data are expressed as z-score intensity expression levels and presented via standard Box and whiskers plot. ****p-value < 0.0001 and *p-value < 0.05 *versus* healthy control group. (**C**) Linear regression analysis of GFAP and GJA1 z-scores in sALS group.

We first tested the hypothesis of a potential role of Cx43 in human ALS analysing the z-score of mRNA expression levels in the central nervous system of control and sporadic (s)ALS patients. We used the NCBI Gene Expression Omnibus (GEO) database (http://www.ncbi.nlm.nih.gov/geo/) to select human healthy and ALS gene expression dataset. We analysed the GFAP (encoding for the glial fibrillary acidic protein) and GJA1 (encoding for Cx43) expression levels in central nervous system biopsies of healthy and sALS patients. Our analysis revealed that in sALS patients both GFAP and GJA1 mRNA levels were significantly increased as compared to the healthy counterpart (Figure 1A, 1B). We then moved to analyse a potential correlation between GFAP and GJA1 performing a linear regression analysis, finding a positive correlation between tested genes in human sALS central nervous system (r^2^ = 0.4765, p-value < 0.0001, Figure 1C).

### CTB-Sap-induced motoneuronal depletion mediates behavioural impairment in mice

**Figure 2:**
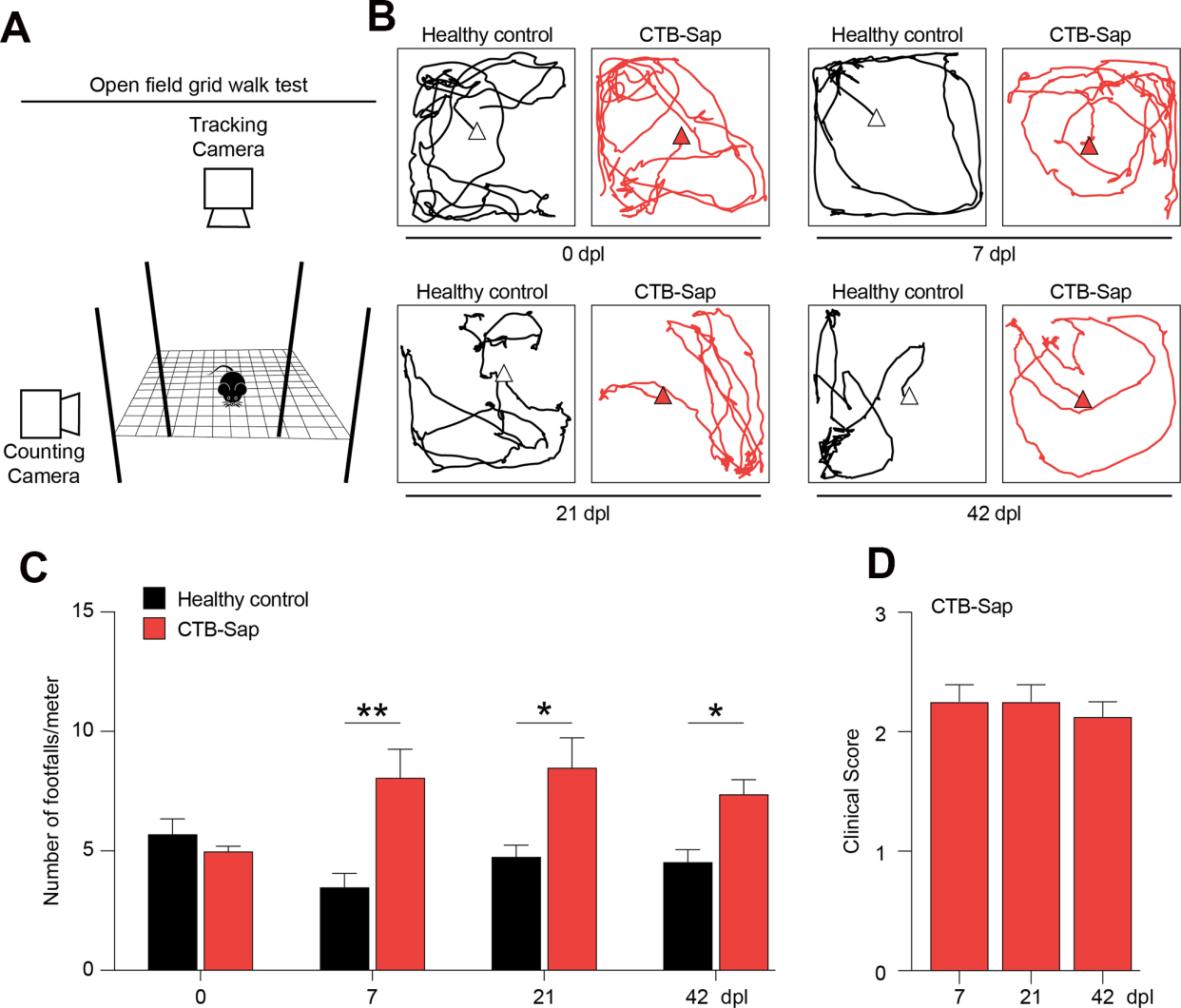
**Motor impairment in spinal motoneuronal depleted CTB-Sap mice.** (**A**) Experimental setting of open field grid walk behavioural platform. (**B**, **C**) Representative tracks (**B**) and quantification of the number of footfalls over meter (**C**) of healthy (black) and CTB-Sap lesioned (red) mice at 0, 7, 21 and 42 days post-lesion (dpl); data are mean ± SEM; **p-value < 0.01 and *p-value < 0.05 versus healthy control group. (**D**) Clinical score of CTB-Sap-lesioned mice in the time course of lesion; data are mean ± SEM.

In order to analyse the effects of motoneuronal loss and its impact on behavioural and neuropathological signs *in vivo*, we established a model of spinal motoneuronal depletion induced by the neuronal targeting toxin CTB-Sap, which is retrogradely transported throughout axons to the spinal cord. We evaluated the behavioural impact of motoneuronal loss at 0, 7, 21 and 42 days post-lesion (dpl), performing an open field grid walk test (Figure 2A), tracking the distance covered by mice during the task with a tracking camera, and the number of footfalls over meter with a counting camera (Figure 2A). We found that both healthy control and CTB-Sap lesioned mice were active in the performance and covered an average distance of 3.2 ± 0.5 and 4.2 ± 1.0 meters, respectively (p-value > 0.444, Figure 2B). We also found that CTB-Sap lesioned mice showed a significant increase of the rate of errors as soon as 7 dpl and that such motor coordination impairment was retained up to 42 dpl (Figure 2C). We confirmed this evidence evaluating the clinical impairment during the time course of disease. Our data indicate that lesioned mice presented a stable impairment and a clinical score of about 2 (Figure 2D), showing leg extension towards the lateral midline and also affected stepping during locomotion test.

### CTB-Sap induces typical electromyographic signs of denervation

**Figure 3:**
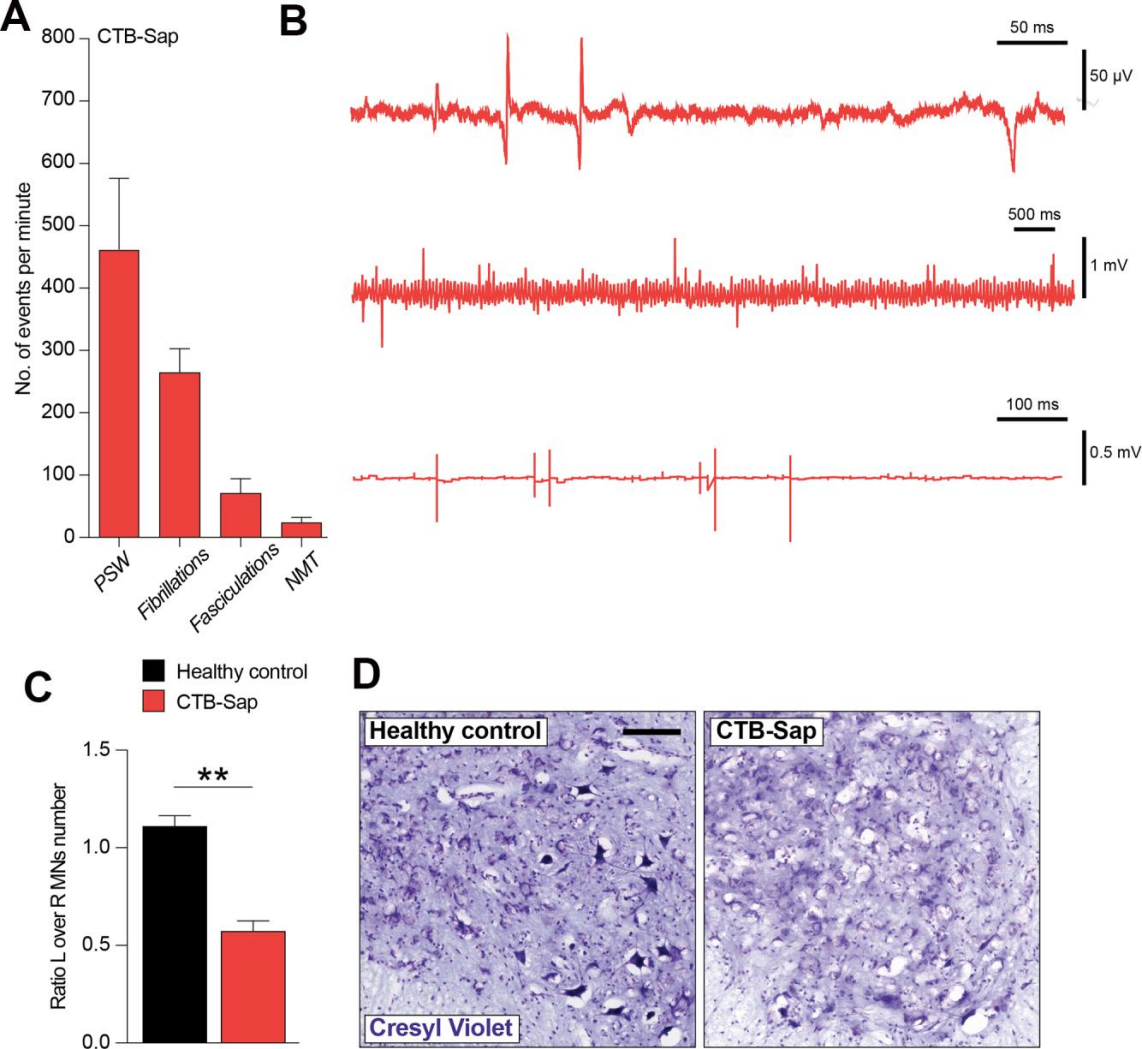
**Electromyographic signs and neuropathological analysis of CTB-Sap lesioned mice.** Quantification (**A**) and representative profile of electromyographic activity of gastrocnemius muscle in CTB-Sap lesioned mice. (**B**) positive sharp waves (PSW), fibrillations, fasciculation and neuromyotonia (NMT); data in (**A**) are expressed as mean events per minute ± SEM. (**C**) Quantification of the number of neurons in left (L) over right (R) ventral horn of CTB-Sap lesioned mice versus healthy control; data are expressed as mean ratio L over R ± SEM; **p-value < 0.01 versus healthy control. (**D**) Representative images of cresyl violet stained motoneurons in left Rexed lamina IX of healthy control and CTB-Sap lesioned mice. Scale bar: 100 μm. MNs: motoneurons.

In order to better characterize the denervation in CTB-Sap-injected mice, we performed an electromyographic recording into the left gastrocnemius muscle to find signs of denervation and spontaneous electrical activity. The results of our analysis are reported in Figure 3A, 3B and show that CTB-Sap induces muscle denervation, as suggested by a relevant number of positive sharp waves, fibrillations, fasciculations and neuromyotonia (Figure 3B). Of note, our electromyographic analysis found no obvious signs of myopathy.

### Spinal cord neuropathological analysis

We then moved to analyse the neuropathological effects of CTB-Sap, by quantifying the impact onto the resident motoneuronal populations. Our analysis revealed a striking reduction of left over right motoneuron number in Rexed lamina IX of CTB-Sap lesioned mice versus healthy control (Figure 3C, 3D). This depletion is also evident in Figure 3D, which shows representative images of cresyl violet-positive motoneurons in left Rexed lamina IX of healthy control and CTB-Sap mice.

### Cx43-mediated coupling in Rexed lamina IX glial cells

**Figure 4:**
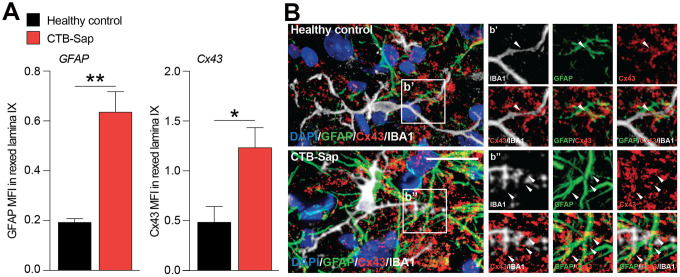
**Increase of Cx43 in the spinal cord cell populations of motoneuron-depleted spinal cord.** (**A**) Quantification of mean fluorescence intensity (MFI) of GFAP and Cx43 in the left lamina IX of healthy control and CTB-Sap lesioned mice; data are expressed as mean ± SEM; **p-value < 0.01 and *p-value < 0.05 versus healthy control. (**B**) Representative confocal images of Cx43 (red) immunofluorescence analysis in lamina IX of healthy control and CTB-Sap lesioned mice; images show also markers for astroglial cells (GFAP, green) and microglia (IBA1, white); scale bar 20 μm.

**Figure 5:**
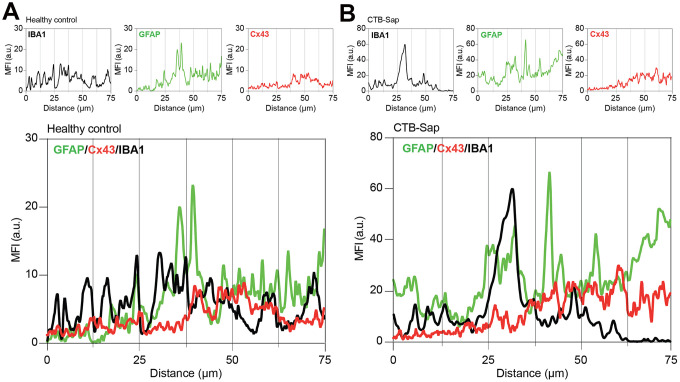
**Cx43-based channels profile in microglial/astroglial milieu in motoneuron-depleted spinal cord.** Profile plot of MFI of IBA1 (black plot), GFAP (green plot), and Cx43 (red plot) and plots overlay (bottom panel) in Rexed lamina IX of healthy control (**A**) and CTB-Sap lesioned mice (**B**); data are MFI arbitrary units (a.u.) of spinal confocal acquisitions.

The relevance of astroglial Cx43 in human ALS prompted us to evaluate a potential involvement of this Cx in a reductionist model of spinal motoneuronal loss induced by CTB-Sap. We assessed Cx43 expression in our model, by measuring the Cx43 mean fluorescence intensity (MFI) in the spinal cord of healthy control and CTB-Sap mice, finding a significant MFI increase in GFAP and Cx43 levels in Rexed lamina IX of motoneuronal depleted spinal cord (Figure 4A, 4B). Such an increase was coupled with morphological changes in astroglial (i.e. GFAP positive) and microglial (i.e. IBA1 positive) cell populations (Figure 4B). Finally, we analysed the profile plot of GFAP, IBA1 and Cx43 in the spinal cord of healthy control (Figure 5A) and CTB-Sap-lesioned (Figure 5B) mice, confirming an increased colocalization between Cx43 and GFAP/IBA1 (Figure 5A, 5B).

## DISCUSSION

It is known that glial cells, both astrocytes and microglia, hold key physiological roles in the central nervous system, such as immunological surveillance, blood brain barrier function, synaptic activity, neuronal trophism and metabolic support [1, 14–18]. In the last decades, advances have come to suggest a critical role of neuroglial cross-talk and related microenvironmental modulation during neurodegenerative disorders [7, 19, 20]. Such a role, besides being an attractive target due to its pathophysiological importance, also opens new scenarios to develop potential effective therapeutic strategies.

Several *in vitro* and *in vivo* models of main neurological conditions such as stroke, multiple sclerosis, Alzheimer’s disease and ALS, demonstrated that reactive astrocytes and microglia amplify neuroinflammation and neurodegeneration through aberrant GJs/HCs communication [21]. It is noteworthy that even in aging models, dysregulation of astroglial population and Cx43 dynamic expression profile may be one of the responsible mechanisms for Aβ deposits in the brain [9, 22, 23].

Notably, an abnormal increase in Cx43 expression has been described as one of the mechanisms for astrocyte-mediated toxicity in both SOD1(G93A) mice and in the central nervous system of ALS patients [20].

Herein, we first analysed available data on NCBI GEO database to select human ALS transcriptome dataset (E-MTAB-2325) in order to verify whether astrogliosis and reactive Cx43 expression, which are both reported in ALS neuropathology, were positively correlated. Such analysis suggested that astrocytes represent the leading cell population in showing Cx43 expression, and that human astroglial reactive Cx43 finds a correspondence in mice model of motoneuronal diseases. Astroglial cells are able to communicate with each other through Cxs-based GJs, mainly expressing Cx43 [7]. This direct astrocyte-to-astrocyte communication is involved in homeostatic processes within the complex intercellular network they form, allowing metabolites, small molecules and second messengers trafficking. During neurodegenerative disease, central nervous system microenvironment is substantially affected by inflammatory cytokines released by reactive microglia also acting on astroglial cells. Astrogliosis and concomitant reactive Cx43 expression contribute to homocellular and heterocellular communication, also releasing reactive oxygen species and inflammatory mediators. Therefore, such unbalanced communication fosters neurotoxic and proinflammatory loop of neurodegenerative disease [24, 25].

We also assessed a toxin-based model of motoneuronal depletion established using CTB-Sap [14, 26, 27], which selectively targets axon terminals and kills motoneurons by retrograde suicide transport [28, 29], thus inducing both muscular denervation and behavioural impairment of motor performance. Our reductionist *in vivo* model of motoneuronal disorders showed functional deficits and electromyographic signs typical of both transgenic ALS mouse model and human ALS patients [30–32]. In particular, our electromyography data revealed that CTB-Sap-induced motoneuronal ablation does not induce myopathy. Indeed, no obvious signs of myopathy were found in motoneuronal depleted mice. In myopathic diseases, in addition to apparent fibrillation potentials and positive sharp waves, normal or early recruitment is found, whereas in our animal model we found profuse fibrillation potentials and positive sharp waves associated with reduced recruitment, that is a typical pattern found in neuropathy and also observed in ALS patients [33, 34].

In CTB-Sap induced motoneuronal depletion, we have therefore observed typical ALS electromyographic signs of denervation, thus supporting this model as a valuable tool to study neurodegeneration and central effects of reduced motoneuronal pool.

A significant aspect of our model is the evidence of reactive astrocytes expressing Cx43, which suggested an increase in intercellular communication. Our evidence does not support a relationship between neuronal ablation efficiency and glial cells activation, although a potential relationship between spared motoneurons modulating the activation and function of both microglia and astrocytes, may occur. Moreover, enhanced Cx43 expression also activates a positive-loop conditioning ventral horn microenvironment that likely exerts a detrimental effect on spared motoneurons. Accordingly, negative effects induced by Cx43 overexpression have been reported in experimental models of ALS, showing that increased glial Cx43-channels significantly affect neuronal activity and wellness [20]. In particular, experimental evidence supports the hypothesis that Cx43 could exert such a detrimental role when assembled as HCs and exposed to cell membrane. Such an effect may be linked to increased excitotoxic calcium release, reactive oxygen species, glutamate and ATP, thus further inducing neuronal distress and death [1, 25, 35–37]. The role of microglial cells during neurodegeneration is also of importance, in particular for their role as master regulators of inflammatory cytokine release. Microglia modulates astroglial functions releasing IL-1β and TNFα that have been linked to an overall increase of Cx43-based HCs activity, further sustaining neuronal suffering [38, 39].

In the present report, we found an altered glial activity in an experimental model of motoneuronal depletion, resulting in a reactive Cx43 expression. Further studies will help to characterize the molecular mediators and the role of selective silencing and/or pharmacological modulation of Cx43 function. GJs- or HCs-forming protein in CTB-Sap induced focal motoneuronal depletion may also offer the opportunity to evaluate a potential discrepancy of Cx43 biological meaning in the early versus the late stage of disease. Crucial information may be derived by Cx43 knockout models upon neurodegenerative insults, even if potential cross-modulation among Cxs may take place. Of note, the role of microglial GJs and HCs is still matter of debate, in particular on the heterocellular (i.e. microglia-astrocytes) GJs composition. A deeper investigation on the role of Cx43 in microglial cell population and on the crucial role of HCs in neuroglial crosstalk will help to elucidate biological substrates and to highlight potential therapeutic targets in neurodegenerative diseases.

## MATERIALS AND METHODS

### Human ALS data

For human ALS data, we used the NCBI Gene Expression Omnibus (GEO) database (http://www.ncbi.nlm.nih.gov/geo/) to select human ALS central nervous system transcriptome dataset (E-MTAB-2325) analysing the GFAP (encoding for the glial fibrillary acidic protein) and GJA1 (encoding for Cx43) expression levels. Mesh terms “central nervous system”, “ALS” and “Human” were used to identify potential datasets of interest. Healthy control tissues were matched for age, post-mortem (PM) delay and central nervous system region. The samples characteristics are available in Table 1. The analysis of microarray data by Z-score transformation was performed using MultiExperiment Viewer (MeV) software (The Institute for Genomic Research (TIGR), J. Craig Venter Institute, USA), in order to allow the comparison of microarray data independent of the original hybridization intensities and reduce the noise of original intensity signal [40–42].

**Table 1:** Characteristics of healthy control and sALS human samples.

**Sample**	**Age**	**Male**	**Female**
**Healthy control**	55.1±14.4	9	1
**ALS**	56.70±9.94	20	11

### Animal model

All experiments were performed in accordance with the principle of the Basel Declaration as well as with the European Communities Council directive and Italian regulations (EEC Council 2010/63/EU and Italian D.Lgs. no. 26/2014). The protocol was approved by the Italian Ministry of Health (auth. no. 1133/2016-PR). All efforts were made to replace, reduce, and refine the use of laboratory animals. Experiments were performed on 8–12 weeks old male 129S2/SvPasCrl (Charles River Laboratories, Calco, Italy), as previously described [13, 14]. Briefly, a total number of 16 animals were used in this study, randomly assigned to the HC group (n = 8) or the CTB-Sap (12 μg injected into the left gastrocnemius muscle) lesioned group (n = 8). For CTB-Sap injection, mice were anesthetized with isoflurane (4% for induction, 2% for maintenance). Mice were then observed for up to 42 days post lesion (dpl) evaluating the clinical score based on the following criteria: 0 = healthy; 1 = collapse or partial collapse of leg extension towards the lateral midline during the tail suspension test; 2 = toes curl under at least twice during walking of 30 cm or any part of the foot is dragging along the cage bottom/table; 3 = rigid paralysis or minimal joint movement, foot not being used for generating forward motion; 4 = mouse cannot straighten itself within 30 s after being placed on either side.

### Electromyography

Electromyographic recording was performed as previously described [14]. Briefly, at 42 dpl mice were anesthetized with isoflurane and CTB-Sap injected gastrocnemius muscle was exposed and examined by a portable two-channel EMG device (Myoquick, Micromed S.p.A., Mogliano Veneto, Treviso, Italy) using 1 bipolar concentric needle electrode inserted in the gastrocnemius and 1 grounded electrode.

### Open field grid walk test

Open field grid walk test was performed at 0, 7, 21, and 42 dpl using a platform equipped with a tracking camera and a counting camera. Animals were placed in the arena and were free to move and to explore during the behavioural test. Each performance was recorded for 2 minutes and matched tracking and counting video were analysed off-line using Ctrax tracker software version 0.5.18 for Mac.

### *Ex vivo* tissue processing

At 42 dpl, spinal cord isolation, cryo-sectioning and immunofluorescence analysis were performed as previously described [43]. Briefly, isolated spinal cords were post-fixed with 4% paraformaldehyde overnight at 4 °C. Samples were then cryo-protected with 30% sucrose in PBS overnight at 4 °C and then embedded in Optimum Cutting Temperature medium. Embedded samples were snap frozen in liquid nitrogen and cut into 20 μm-thick cryosections. Sections were collected on SuperFrost slides and stored at - 80 °C until use. Before performing experiments, sections were dried at room temperature for 45 minutes and then washed in deptH_2_O and PBS 2 times for 5 minutes at room temperature.

### Cresyl violet

For cresyl violet staining, spinal cord sections were dehydrated with increasing ethanol (70%, 95% 100%) in deptH_2_O for 3 minutes and then in xylene for 5 minutes. Dehydrated sections were then homogenously rehydrated and stained with a solution of 0.2% sodium acetate, 1% cresyl violet, 3% glacial acetic acid in deptH_2_O for 10 min at room temperature. Sections were then washed in water, dehydrated in increasing ethanol concentrations, clarified in xylene and coverslipped.

### Immunofluorescence

Immunofluorescence was performed as previously described [43–46]. Briefly, samples were incubated overnight at 4 °C with mouse monoclonal anti-GFAP (BD Biosciences, Cat# 610566, RRID: AB_397916, 1:500), rabbit polyclonal anti-Cx43 (Cell Signaling Technology, Cat# 3512, RRID: AB_2294590, 1:200), goat polyclonal anti-IBA1 (Novus Biologicals, Cat# NB100-1028, RRID: AB_521594, 1:500). The following day, sections were washed in 0.1% Triton X-100 in PBS 3 times at room temperature and then incubated 1 hr at room temperature with appropriate combination of secondary antibodies: goat polyclonal anti-mouse (Alexa Fluor 488, Thermo Fisher Scientific, Cat# A-11001, RRID: AB_2534069, 1:1’000), goat polyclonal anti-rabbit (Alexa Fluor 564, Molecular Probes, Cat# A-11010, RRID: AB_143156, 1:1’000) and donkey anti-goat (Alexa Fluor 647, Thermo Fisher Scientific, Cat# A-21447, RRID:AB_2535864). Nuclei were counterstained with DAPI (1:10’000, Invitrogen) for 5 min at room temperature and then mounted with BrightMount mounting medium (Abcam). Profile plots for immunofluorescence images were obtained as previously described [43].

### Statistical analysis

All tests were performed in GraphPad Prism (version 5.00, GraphPad Software) or RStudio (version 1.0.153, RStudio Inc.). Data were tested for normality using a D’Agostino and Pearson omnibus normality test and subsequently assessed for homogeneity of variance. Data that passed both tests were further analyzed by two-tailed unpaired Student’s t-test for comparison of n = 2 groups. For comparison of n &ge; 3 groups, one-way or two-way ANOVA was used where appropriate, and associations between variables were analysed by linear regression and correlation.
